# Hydrosilylation vs. Piers–Rubinsztajn: Synthetic Routes to Chemically Cross-Linked Hybrid Phosphazene-Siloxane 3D-Structures

**DOI:** 10.3390/polym17141967

**Published:** 2025-07-17

**Authors:** Andrey S. Esin, Anna I. Chernysheva, Ekaterina A. Yurasova, Ekaterina A. Karpova, Vyacheslav V. Shutov, Igor S. Sirotin, Mikhail A. Soldatov, Mikhail V. Gorlov, Oleg A. Raitman

**Affiliations:** 1 Department of Chemical Technology of Plastic Materials, Mendeleev University of Chemical Technology of Russia, Miusskaya Square 9, 125047 Moscow, Russia; esin.a.s@muctr.ru (A.S.E.); anna.iv.chernysheva@gmail.com (A.I.C.); karpova.e.a@muctr.ru (E.A.K.); sirotin.i.s@muctr.ru (I.S.S.); 2 Department of Plastic Processing Technology, Mendeleev University of Chemical Technology of Russia, Miusskaya Square 9, 125047 Moscow, Russia; yekaterina.yurasova@yandex.ru (E.A.Y.); shutov.v.v@muctr.ru (V.V.S.); 3 Department of Chemical Technology of Polymer Composite Paint Materials and Coatings, Mendeleev University of Chemical Technology of Russia, Miusskaya Square 9, 125047 Moscow, Russia; soldatov.m.a@muctr.ru; 4 Department of Physical Chemistry, Mendeleev University of Chemical Technology of Russia, Miusskaya Square 9, 125047 Moscow, Russia

**Keywords:** phosphazenes, cross-linked structures, hydrosilylation, Piers–Rubinsztajn reaction, eugenol, siloxanes, polymer matrix, model compounds, hybrid, siloxane-phosphazene

## Abstract

Exploration of new ways for the direct preparation of cross-linked structures is a significant problem in terms of materials for biomedical applications, lithium batteries electrolytes, toughening of thermosets (epoxy, benzoxazine, etc.) with interpenetrating polymer network, etc. The possibility to utilize hydrosilylation and Piers–Rubinsztajn reactions to obtain cross-linked model phosphazene compounds containing eugenoxy and guaiacoxy groups has been studied. It was shown that Piers–Rubinsztajn reaction cannot be used to prepare phosphazene-based tailored polymer matrix due to the catalyst deactivation by nitrogen atoms of main chain units. Utilizing the hydrosilylation reaction, a series of cross-linked materials were obtained, and their properties were studied by NMR spectroscopy, FTIR, DSC, and TGA. Rheological characterizations of the prepared tailored matrices were conducted. This work showed a perspective of using eugenoxy functional groups for the preparation of three-dimensional hybrid phosphazene/siloxane-based materials for various applications.

## 1. Introduction

The rapid development of technological progress demands the preparation of novel and effective current sources, which can work in a wide temperature range and possess high charge capacity and rechargeability [1,2,3]. Among all polymers used for lithium-ion batteries, poly(ethylene oxide) (PEO) is one of the most studied, which possesses good ion conductivity due to the high flexibility of the polymeric chain and its ability to coordinate lithium ions with oxygen atoms [4]. The main disadvantage of this polymer is its high tendency for crystalline phase formation, which in turn leads to a decrease in ion conductivity [5]. On the other hand, phosphazene polymers are good candidates for polyelectrolyte materials because of their high flexibility of the polymeric chain, high chemical and thermal stability, biocompatibility, fire-proofing properties, and easy backbone derivatization [6,7,8], especially after the convenient preparation methods for trichloro(trimethylsilyl)phosphoranimine Cl_3_P=NSiMe_3_ [9,10,11] and its further living cationic polymerization were discovered [12,13]. It is worth noting that phosphazene chains can also contribute to metal cations’ coordination due to the possibility of their interaction with a lone pair of backbone nitrogen atoms [7,14]. The most known efficient phosphazene alternative for PEO is poly(methoxyethoxyethoxy)phosphazene (PMEEP)—a fully amorphous polymer [5,15,16]. Despite a number of obvious benefits, like a very low glass transition temperature and 6 oxygen atoms per 1 chain unit, PMEEP possesses quite low dimensional stability. One route to overcome this drawback is cross-linking, which, by the way, can also prevent the formation of lithium dendrites during the charging/discharging processes [17]. Phosphazene cross-linking is traditionally performed using Co^60^ radiation through C-H bonds cleavage, but the process is not well controlled and can yield a matrix that is too hard. At the same time, the formation of a rigid tailored network will decrease the segmental motion and, as a result, ionic conductivity. So, for better conductive properties of cross-linked polymeric electrolytes, the cross-linking units are to be quite flexible and do not influence segmental mobility. In light of this, highly flexible functional oligosiloxanes seem to be ideal cross-linkers [18,19,20]. On the other hand, eugenol fragments are promising functional groups to be introduced into the polyphosphazene structure [21,22,23]. Eugenoxy fragments on phosphorus atoms have two active centers, which can react with hydride-containing siloxanes in different ways: allylic groups through the hydrosilylation and methoxy groups through the Piers–Rubinsztajn reaction [24]. So, as no one has used the Piers–Rubinsztajn for cross-linking of phosphazene compounds, it seems to be interesting to study this opportunity and compare it with the well-known hydrosilylation method. In this work, we have synthesized model compounds hexaeugenoxycyclotriphosphazene and hexaguaiacoxycyclotriphosphazene and studied their ability to cross-link with hydride-terminated oligosiloxanes.

## 2. Materials and Methods

### 2.1. Materials

Eugenol (≥98% pure, Acros Organics, Geel, Belgium) and guaiacol (≥99% pure, Acros Organics) were distilled prior to use. Hexachlorocyclotriphosphazene was obtained from Rushim (Moscow, Russia) and purified by vacuum sublimation. Sodium metal and hydride-terminated siloxane compounds HSi(CH_3_)_2_OSi(CH_3_)_2_H (TMDS), HSi(CH_3_)_2_(Si(CH_3_)_2_O)_6_Si(CH_3_)_2_H (Si6), and HSi(CH_3_)_2_(Si(CH_3_)_2_O)_30_Si(CH_3_)_2_H (Si30) were purchased from Sigma-Aldrich Corp. (St. Louis, MO, USA) and used as received.

Karstedt catalyst Pt_2_dvs_3_ (toluene solution, C_Pt_ = 1 mg/mL), obtained from Penta-91 (Moscow, Russia), and tris(pentafluorophenyl)borane B(C_6_F_5_)_3_, purchased from P&M-invest (Moscow, Russia), were used without any additional purification.

Solvents (dioxane, toluene) were purified according to the known methods [25] and were used as freshly distilled. The water content was controlled in ppm by a Metrohm 899 Coulometer. All reactions were carried out under an inert atmosphere of dry nitrogen using standard Schlenk techniques.

### 2.2. Characterization Methods

^31^P and ^1^H NMR spectra were recorded by a Bruker Avance III 400 spectrometer operating at frequencies 162 and 400 MHz, respectively. The spectra were recorded in CDCl_3_ and reported in parts per million (δ) relative to the residual solvent signal for ^1^H (7.26 ppm) spectra, and 85% H_3_PO_4_ (0.0 ppm) as an external standard for ^31^P NMR spectra.

FTIR spectra were measured with the use of a Nicolet iS50 (Thermo Fisher Scientific, Waltham, MA, USA) spectrometer in ATR mode with a diamond crystal.

Differential scanning calorimetry (DSC) was performed on a Netzsch DSC 204 F1 Phoenix instrument (Netzsch, Selb, Germany) in a nitrogen atmosphere (20 mL/min) at a heating rate of 20 deg/min on samples weighing ~5–8 mg.

Thermal gravimetric analysis (TGA) was performed on Derivatograph-1500Q at a heating rate of 20 deg/min in an air atmosphere.

The rheological properties of compositions were measured using an MCR 302 rotational rheometer (Anton Paar, Graz, Austria). In these experiments, a plate–plate operating unit with the diameter of a rotating plate of 25 mm was used. The dynamic tests of the samples were carried out in the frequency range of 0.1–100 Hz with a constant oscillation amplitude of 5%, which ensured the strain of the samples was within the region of linear viscoelasticity. The temperature dependence of the modules was obtained in the temperature range from 5 to 100 degrees Celsius at a constant frequency of 1 Hz and at a constant oscillation amplitude of 5%.

### 2.3. Synthesis of Cyclic Phosphazene P_3_N_3_Gua_6_ or P_3_N_3_Eug_6_

An amount of 15.70 g (0.1264 mol) of guaiacol, in the case of P_3_N_3_Gua_6_, or 20.76 g (0.1264 mol) of eugenol, in the case of P_3_N_3_Eug_6_, and 150 mL of dioxane were charged into the three-necked flask, equipped with a magnetic stirrer and reflux condenser. Then, 2.64 g (0.1149 mol) of sodium was added in the form of thin sliced plates, and the mixture was stirred at 70 °C until the full dissolution of sodium. A solution of 5.00 g (0.0144 mol) of hexachlorocyclotriphosphazene in 50 mL of dioxane was added dropwise, and the reaction mixture was stirred at 70 °C for 24 h, and then poured into the excess water. The formed precipitate was dissolved in chloroform, washed with water, and the resulting solution was dried over Na_2_SO_4_. Then, chloroform was rotary evaporated, and the product was dissolved in a mixture of 30 mL of dichloromethane and 30 mL of ethanol. After long-lasting dichloromethane evaporation, a yellow crystalline product was formed, which was then dried at 60 °C under vacuum for 4 h. Yields: 9.82 g (78%) of P_3_N_3_Gua_6_ and 10.97 g (69%) of P_3_N_3_Eug_6_.

### 2.4. Preparation of Hybrid Cross-Linked Phosphazene-Siloxane Materials

An amount of 0.2 g of eugenoxy-substituted phosphazene trimer was dissolved in toluene. Then, a catalyst B(C_6_F_5_)_3_ in toluene (40 mg/mL) or Karstedth catalyst solution in toluene (C_Pt_ = 1 mg/mL) and tetramethyldisiloxane HSi(CH_3_)_2_OSi(CH_3_)_2_H or hydride-terminated oligodimethylsiloxane HSi(CH_3_)_2_(Si(CH_3_)_2_O)_n_Si(CH_3_)_2_H, where n = 6 or 30, was added. Then, the mixture was cured for 2 h at 80 °C. During this process, the polymer was crosslinked and dried simultaneously. More detailed data about catalyst, solvent, and siloxane amounts are shown in Table 1. For rheological characterizations, circles with a radius equal to the radius of the measuring plane, i.e., 25 mm, were cut from the prepared samples.

## 3. Results and Discussion

**Scheme 1:**
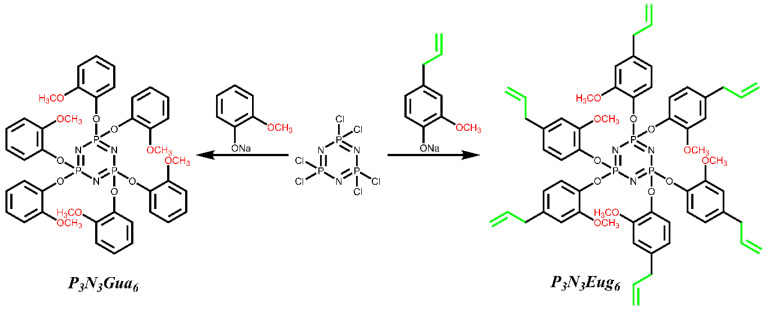
Synthesis of P_3_N_3_Gua_6_ and P_3_N_3_Eug_6_.

First, cyclotriphosphazenes containing eugenoxy or guaiacoxy groups were synthesized (Scheme 1). The reaction between hexachlorocyclotriphosphazene and the sodium salt of eugenol or guaiacol was carried out in a medium of dioxane as a solvent at 70 °C.

**Figure 1:**
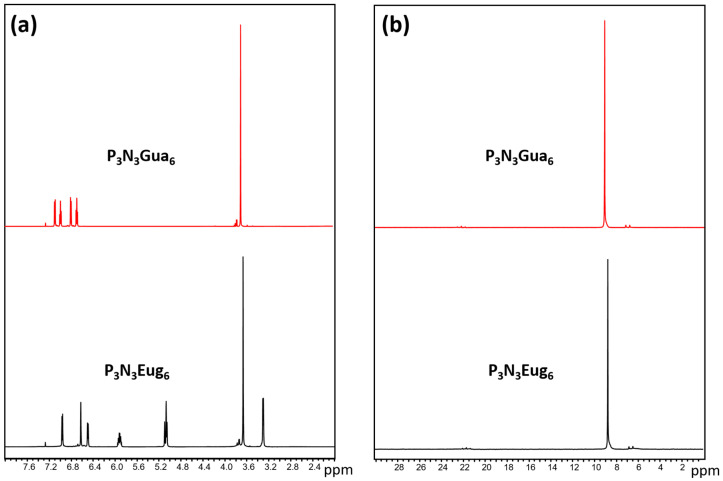
^1^H (**a**) and ^31^P (**b**) NMR spectra of P_3_N_3_Gua_6_ and P_3_N_3_Eug_6_.

The structure of the product was confirmed by NMR spectroscopy (Figure 1).

In the ^1^H spectrum, signals at 6.4–7 and 3.7 ppm are attributed to protons of aromatic and methoxy groups, respectively. Signals of the allyl group at 3.3, 5.0, and 5.9 ppm can be observed as well, which fully corresponds to the literature data [23]. In the ^31^P spectrum, signals at 9.0 and 8.7 ppm stand for P_3_N_3_Gua_6_ and P_3_N_3_Eug_6_, respectively. Other peaks in the ^31^P spectrum, a triplet at 21.9 ppm and doublet at 6.6 ppm, are low-intensity and attributed to the incompletely substituted cyclotriphosphazene compound P_3_N_3_ClEug_5_.

**Scheme 2:**
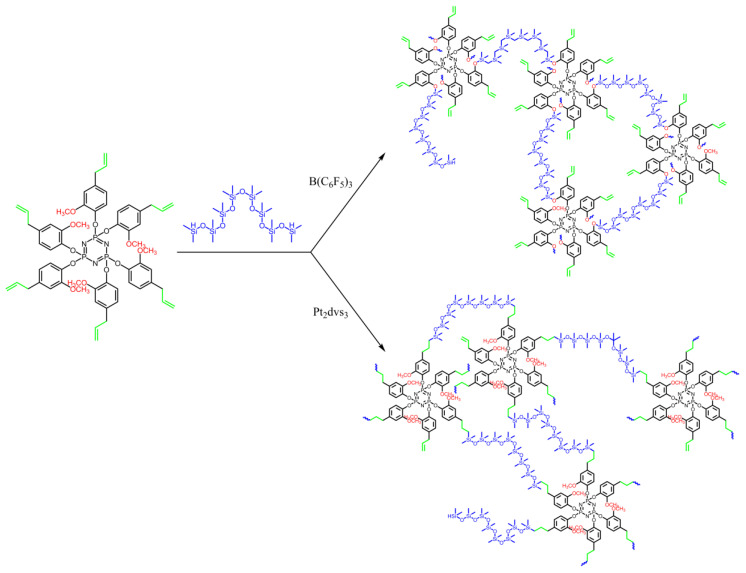
Proposed cross-linking of P_3_N_3_Eug_6_ by means of hydrosilylation and Piers–Rubinsztajn reaction.

The prepared fully derivatized products were planned to undergo the reactions with hydrosilyl groups by Piers–Rubinsztajn and hydrosilylation processes (Scheme 2).

It should be noted here that the most suitable solvents for carrying out these reactions are non-polar toluene and hexane. However, in these media, the hexaguaiacoxycyclophopshazene turned out to be almost completely insoluble, and therefore, attempts were made to carry out the reaction in bulk, as well as in polar THF. Unfortunately, both variants did not give positive results: in the absence of a solvent, phosphazene turned out to be incompatible with siloxane, and in polar THF, despite the satisfactory solubility of the product, hydrosilylation and Piers–Rubinsztajn reaction did not proceed. Thus, further studies were carried out only with a hexaeugenoxy derivative.

In the first step, a hexaeugenoxycyclotriphosphazene was used as a model compound for finding out the optimal reaction conditions during the interaction with hydride-terminated dimethylsiloxane. For the Piers–Rubinsztajn reaction, it was found that no reaction takes place no matter what conditions are supported. This can be explained by the deactivation of the Lewis acid catalyst with nitrogen atoms of phosphazene units, exhibiting Lewis base properties [26], on the one hand, or due to the sterical hindrances for methoxy groups on the other hand.

To confirm the latter assumption, hexa(p-methoxyphenoxy)cyclotriphosphaze with a methoxy group in the para position was synthesized using a known method [27] and subjected to further interaction with hydride-terminated dimethylsiloxane in the presence of B(C_6_F_5_)_3_. However, in this case, the Piers–Rubinsztajn reaction was not observed, which rejects the assumption of steric hindrance. At the same time, the formation of an adduct that is inactive in the Piers–Rubinsztajn reaction is confirmed by both literary data [28] and ^11^B NMR spectroscopy data: there are significant differences in the spectra of pure tris(pentafluorophenyl)borane and its mixture with the phosphazene trimer.

On the next step, hydrosilylation was used. To study the influence of the reaction conditions on the final materials’ properties, we varied parameters such as catalyst amount, concentration of initial P_3_N_3_Eug_6_ in toluene, siloxane chain length, and molar ratio between P_3_N_3_Eug_6_ and cross-linking agent (Table 1).

**Figure 2:**
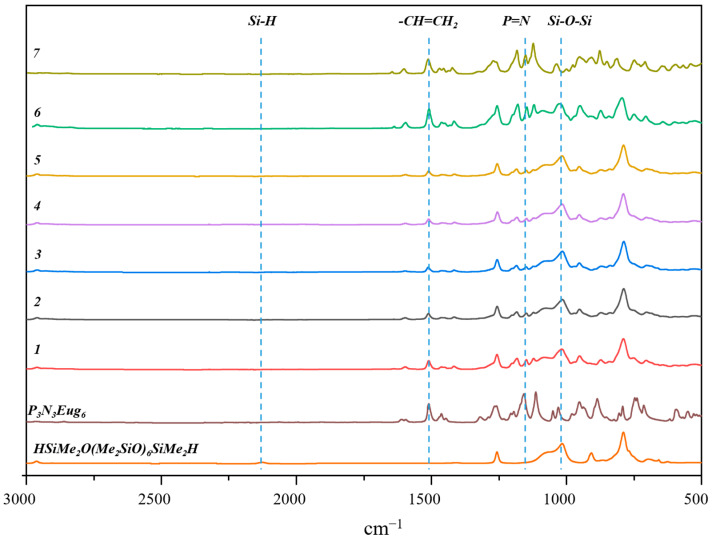
FTIR spectra of initial monomers and cross-linked polymers.

The FTIR spectra (Figure 2) show that the change in these conditions influences cross-linking degree, which can be evaluated by the intensity of the peak at 1510 cm^−1^, attributed to allyl groups’ double bonds. So, in a row of polymers from 1 to 3, the intensity of this peak decreases along with the increase in the catalyst amount used. For polymers 7, 6, and 5, the intensity decreases as the molar ratio P_3_N_3_Eug_6_: siloxane changes from 1:1 to 1:3. In the case of low-cross-linking-degree polymers, the films formed turn out to be either sticky or soluble in organic solvents. When TMDS is used as a cross-linker, the formation of soluble resin, i.e., lower cross-linking degree, can be due to stoichiometric imbalance, caused by rapid evaporation of TMDS (b.p.= 70 °C) under the curing conditions [29].

The disappearance of the peak in the region of 2100 cm^−1^, attributed to the SiH-group, also indicates the success of the reaction. This peak is observed in the spectrum of the initial hydride-terminated siloxane and is absent in the spectra of all cross-linked polymer samples.

**Figure 3:**
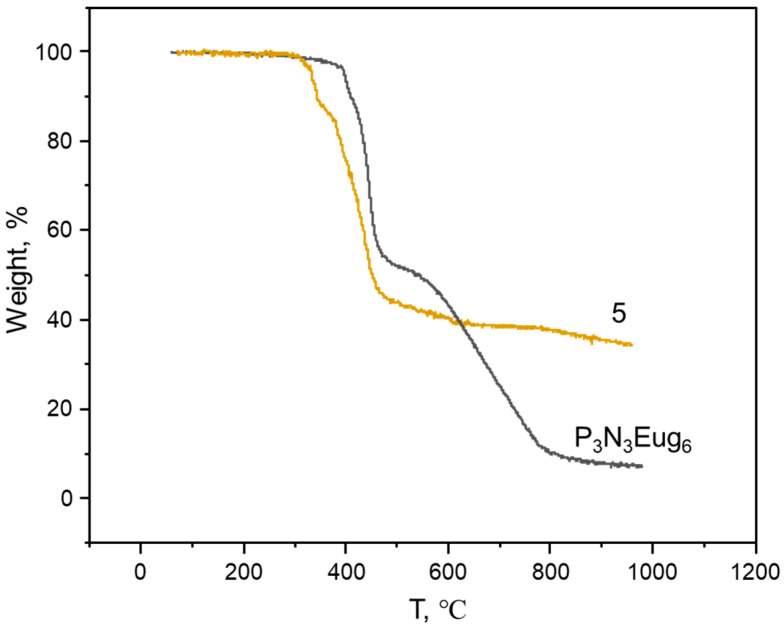
TGA curves of P_3_N_3_Eug_6_ and polymer 5.

Thermal properties of the products obtained were studied by the TGA method (Figure S1 and Table S1), and typical curves for P_3_N_3_Eug_6_ and polymer 5, as a representative sample forming a non-sticky film, are presented in Figure 3. It was shown that polymers had a temperature of 5% mass loss (T_d5_) in the range of 280 to 370 °C, depending on the chemical structure. The lower T_d5_, in comparison with the initial phosphazene monomer P_3_N_3_Eug_6_ (397 °C), is a result of the presence of flexible siloxane units and possible content of residual toluene and non-reactive siloxane monomer. On the other hand, all the polymers possess a higher char yield at 800 °C (17–38% in comparison with 10% for P_3_N_3_Eug_6_), which is explained by the cross-linked structure of the investigated polymers.

According to data from Table 1, the glass transition temperature increases with the decrease in the cross-linking degree. For example, polymer 7 with a lower cross-linking degree has a higher T_g_ (−4.8 °C) in comparison with polymer 5 (−7.5 °C) and 6 (−5.3 °C). The same situation is also observed for polymers 1, 2, and 3. There may be two reasons for such phenomena. First, more cross-linked polymers can contain higher amounts of entrapped residual toluene, which can serve as a plasticizing agent. Second, in highly cross-linked polymers, segmental mobility is provided mostly by flexible siloxane units. When the cross-linking degree decreases, the hard cyclophosphazene units can also lose segmental mobility to some extent, and so the glass transition temperature will increase.

Since the similar cross-linked phosphazene-based materials are supposed to be used in lithium batteries being compressed between two electrodes, they are to be dimensionally stable. In this view, it was interesting to study their mechanical properties with the use of a rotational rheometer [30]. For this characterization, polymers 2, 3, 5, and 9 were used since they gave the best films (non-sticky films in Table 1).

The study of cross-linking processes is of the utmost interest. However, in our case, cross-linking occurs simultaneously with the formation of a film, accompanied by solvent evaporation. A significant volume decrease of the system during the cross-linking process does not allow us to use the known rheometry method. Therefore, we tried to study some rheological aspects of the obtained films, which are the end products of the cross-linking reaction. The films were dried and then analyzed by the method of oscillating rheometry.

In cases when viscoelastic material properties are needed, it is most convenient to measure and describe such characteristics in terms of periodic shear or oscillatory rheology. In periodic shear mode, the sample is imparted with strain, which changes according to the harmonic law:

(1) $$\gamma^{*}=\gamma_{0}\sin\left(\omega t\right).$$

Here, γ* is a complex strain, *γ_0_* is an oscillation amplitude, and *ω* is an angular frequency of oscillation. The resulting response of the material (complex stress σ*) is measured as follows:

(2) $$\sigma^{*}=\sigma_{0}\sin\left(\omega t+\delta \right),$$

where *δ*—loss factor.

When complex notation is used to describe the viscoelastic properties of the material, it is convenient to characterize such properties by the complex shear modulus G*:

(3) $$G^{*}=\frac{\sigma^{*}}{\gamma^{*}}$$

where σ*—complex shear stress, Pa; and γ*—complex shear strain, dimensionless.

The complex modulus, like any other complex value, can be divided into two parts—the real (or storage) modulus and the imaginary (or loss) modulus:

(4) $$G^{*}=G^{\prime }+iG^{\prime \prime }$$

**Figure 4:**
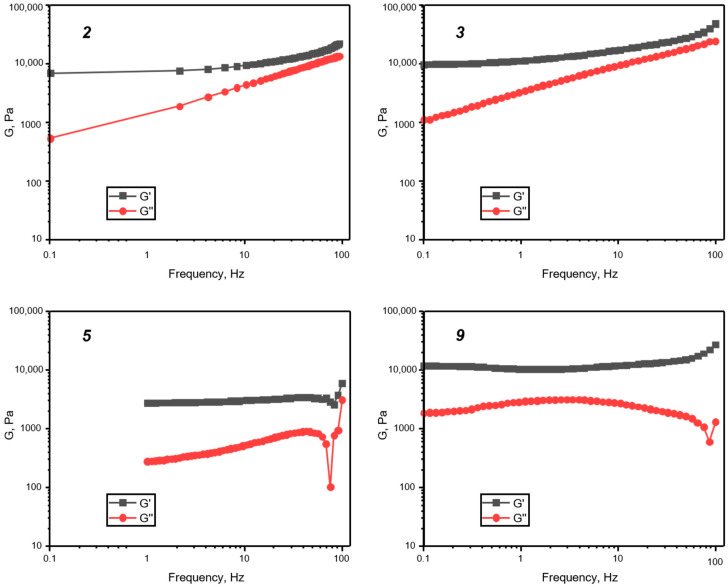
Frequency dependencies of the viscoelastic properties for the films prepared from polymers 2, 3, 5, and 9.

The rheological tests were performed in normal force-controlled mode at 1 N. An amplitude of 1% was selected to perform subsequent frequency sweep tests in the linear viscoelastic region (LVER). Measurements were made over a range of oscillation frequencies at a constant oscillation amplitude and temperature for 4 types of compositions. After solvent evaporation and a simultaneously occurring chemical reaction, the end product was a structured system in the form of a film, which was the actual object of the rheological analysis. Ideal networks have an almost purely elastic response, with the storage modulus $G^{\prime }$ being much higher than the loss modulus $G^{\prime \prime }$, regardless of the frequency. In networks with imperfections, the reaction of the polymer will be frequency dependent as both moduli increase along with the frequency of oscillation. Obtained frequency dependencies of the viscoelastic properties of the films are given below (Figure 4).

As pictured in the diagrams, for all tested samples in the entire frequency range, the value of the storage modulus is higher than the loss modulus and almost independent or slightly dependent on frequency, as it should be for a perfect network system. This indicates that all these samples are in a rubbery state with a good elastic response and the material behaves more like a solid than a viscous liquid: samples 5 and 9—an almost perfect network, and 2 and 3—a network with some imperfections caused by the presence of residual solvent and siloxane or incomplete cross-linking.

From the obtained frequency sweep for samples 2 and 3, it can be seen that at low frequencies, the elastic modulus approaches a kind of plateau value, which is known as a network equilibrium modulus $G_{e}$. The $G_{e}$ value reflects only the chemical cross-links in the network structure since the lifetime of physical entanglements is relatively shorter than the oscillation period. At higher frequencies, the polymer chain entanglements begin to contribute to the material response, increasing both $G^{\prime }$ and $G^{\prime \prime }$ moduli, which indicates a greater mobility of polymer chains and the presence of an incomplete or weak network. A further increase in frequency leads to almost overlapping moduli because the material response in that case is mostly determined by local interactions between polymer chains and physical entanglements that are indistinguishable from chemical cross-links.

Despite the fact that the frequency dependencies for samples 2 and 3 are the same, the equilibrium modulus $G_{e}$ for sample 3 turned out to be higher (9600 Pa for sample 3 vs. 6700 Pa for sample 2), which should be due to the larger amount of a crosslinking agent.

Samples 5 and 9 behave more like ideal networks and solid-like materials since both moduli are slightly dependent on frequency, and no crossover (solid–fluid transition) is observed. Equilibrium moduli for samples 5 and 9 are equal to 2700 and 11800 Pa, respectively. This may indicate a lower mobility of the polymer chains and minimal contribution of physical-type links to the movement of the chains at higher frequencies, which is typical for high cross-link density.

**Figure 5:**
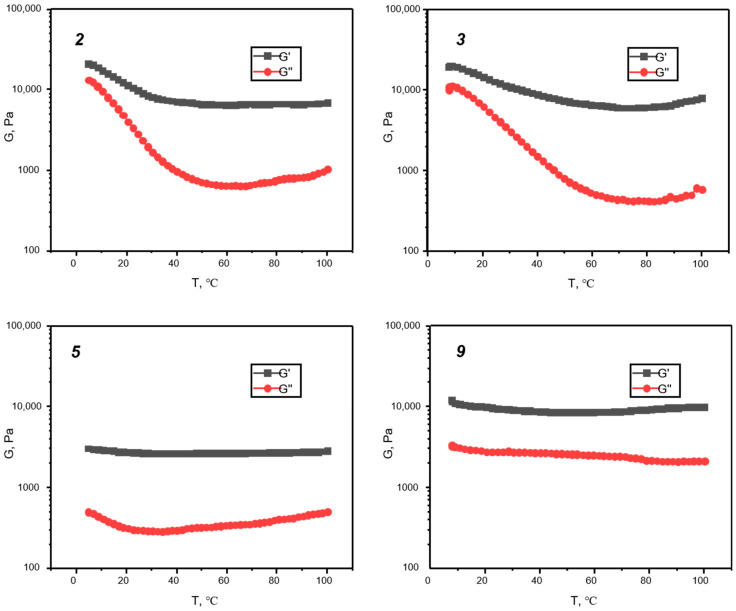
Temperature dependencies of the viscoelastic properties for the films prepared from polymers 2, 3, 5, and 9.

Temperature dependencies of the moduli (Figure 5) indicate that samples 2 and 3 are more sensitive to heat, whereas samples 5 and 9 almost do not respond to a temperature increase. Shear modulus values for samples 2 and 3 fall quite smoothly with the achievement of a plateau, which can be explained by the increasing network segment’s mobility as the temperature rises. However, no moduli crossover indicates that there is no “solid–liquid” transition, and so even fewer cross-linked samples remain elastic.

## 4. Conclusions

In conclusion, we have studied the possibility of cross-linking of phosphazene compounds, having eugenoxy and guaiacoxy groups. It was shown that the Piers–Rubinsztajn reaction is not the appropriate method due to the deactivation of the Lewis acid catalyst with the nitrogen atoms of phosphazene units. On the other hand, the hydrosilylation reaction can be used for the preparation of hybrid phosphazene/siloxane-based polymeric materials with fine-tunable properties. These results open a new perspective on the preparation of novel functional phosphazene-based cross-linked structures for such applications as lithium batteries, dentistry, biomedical applications, elastomers, etc.

## Figures and Tables

**Table 1:** Loadings for the preparation of cross-linked materials based on P_3_N_3_Eug_6_ via hydrosilylation.

# of Sample	Siloxane	Molar Ratio P_3_N_3_Eug_6_: Siloxane	Catalyst Amount, mol. %	Concentration of P_3_N_3_Eug_6_ in Toluene, mg/mL	Result	T_g_, °C
1	Si6	1:3	0.0015	50	Yellow sticky film *	5.3
2	Si6	1:3	0.003	50	Yellow non-sticky film	−5.1
3	Si6	1:3	0.006	50	Yellow non-sticky film	−9.2
4	Si6	1:3	0.003	25	Yellow sticky film	−13.7
5	Si6	1:3	0.003	100	Yellow non-sticky film	−7.5
6	Si6	1:2	0.003	100	Yellow sticky film	−5.3
7	Si6	1:1	0.003	100	Yellow soluble resin	−4.8
8	TMDS	1:3	0.003	100	Yellow soluble resin	8.6
9	Si30	1:3	0.003	100	Yellow non-sticky film	−51.6

* No cross-linking was observed after 2 h of curing at 80 °C. The cross-linked film was formed after 5 h of curing.

## Data Availability

The original contributions presented in this study are included in the article and Supplementary Material. Further inquiries can be directed to the corresponding author.

## Supplementary Materials

The following supporting information can be downloaded at: https://www.mdpi.com/article/10.3390/polym17141967/s1, Table S1: Thermal stability of the obtained polymers; Figure S1: TGA curves for initial P_3_N_3_Eug_6_ and polymers **1**–**9**; Figure S2: ^11^B NMR spectra of tris(pentafluorophenyl)borane (A) and equimolar mixture of tris(pentafluorophenyl)borane with hexachlorocyclotriphosphazene (B), CDCl_3_; Figure S3: ^31^P NMR spectra of hexa(p-methoxyphenoxy)cyclotriphosphazene, CDCl_3_.
