# Supplementary material for: Hydrosilylation vs. Piers–Rubinsztajn: Synthetic Routes to Chemically Cross-Linked Hybrid Phosphazene-Siloxane 3D-Structures
Source: Polymers (Basel). 2025 Jul 17;17(14):1967. doi: 10.3390/polym17141967 (PMC12297966; doi:10.3390/polym17141967)
Supplement: Supplementary file 1 [file polymers-17-01967-s001.zip › polymers-3706647-supplementary.pdf]

Supplementary Materials

Table S1. Thermal stability of the obtained polymers

Sample	T_{d5}, °C	Char yield at 800 °C, % wt.
$P_3N_3Eug_6$	397	10
1	344	38
2	323	34
3	317	28
4	287	19
5	332	38
6	347	35
7	313	30
8	283	18
9	372	17

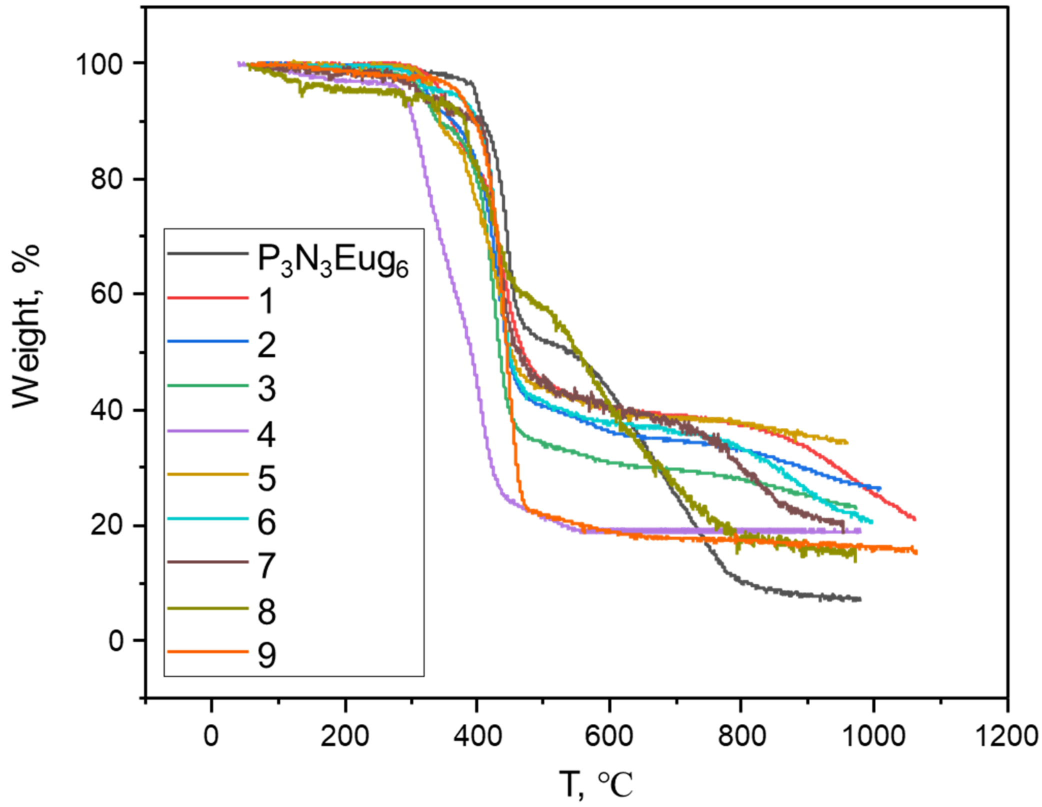

Figure S1. TGA curves for initial $P_3N_3Eug_6$ and polymers ***1-9***

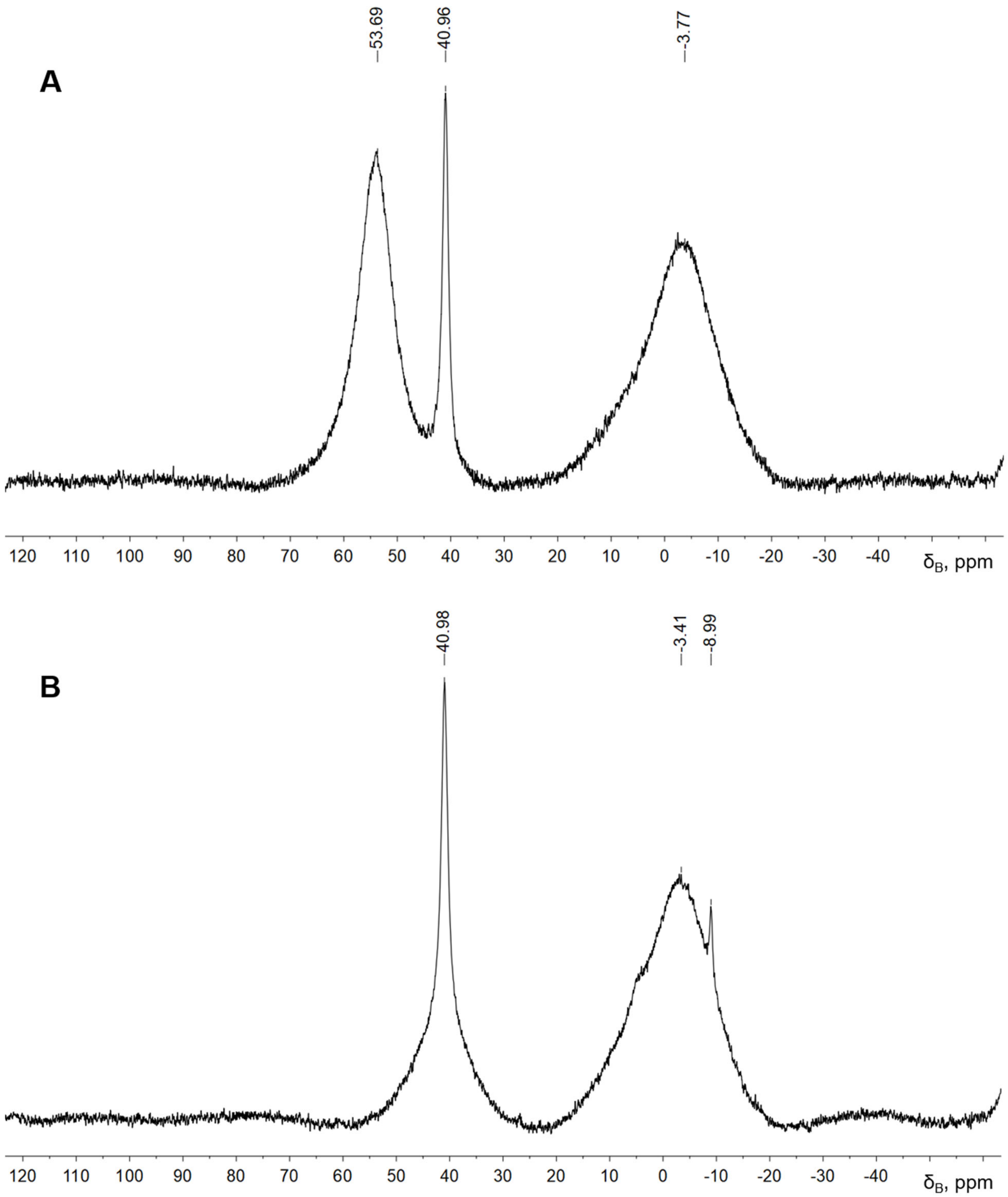

Figure S2. ^{11}B NMR spectra of tris(pentafluorophenyl)borane (A) and equimolar mixture of tris(pentafluorophenyl)borane with hexachlorocyclotriphosphazene (B), $CDCl_3$

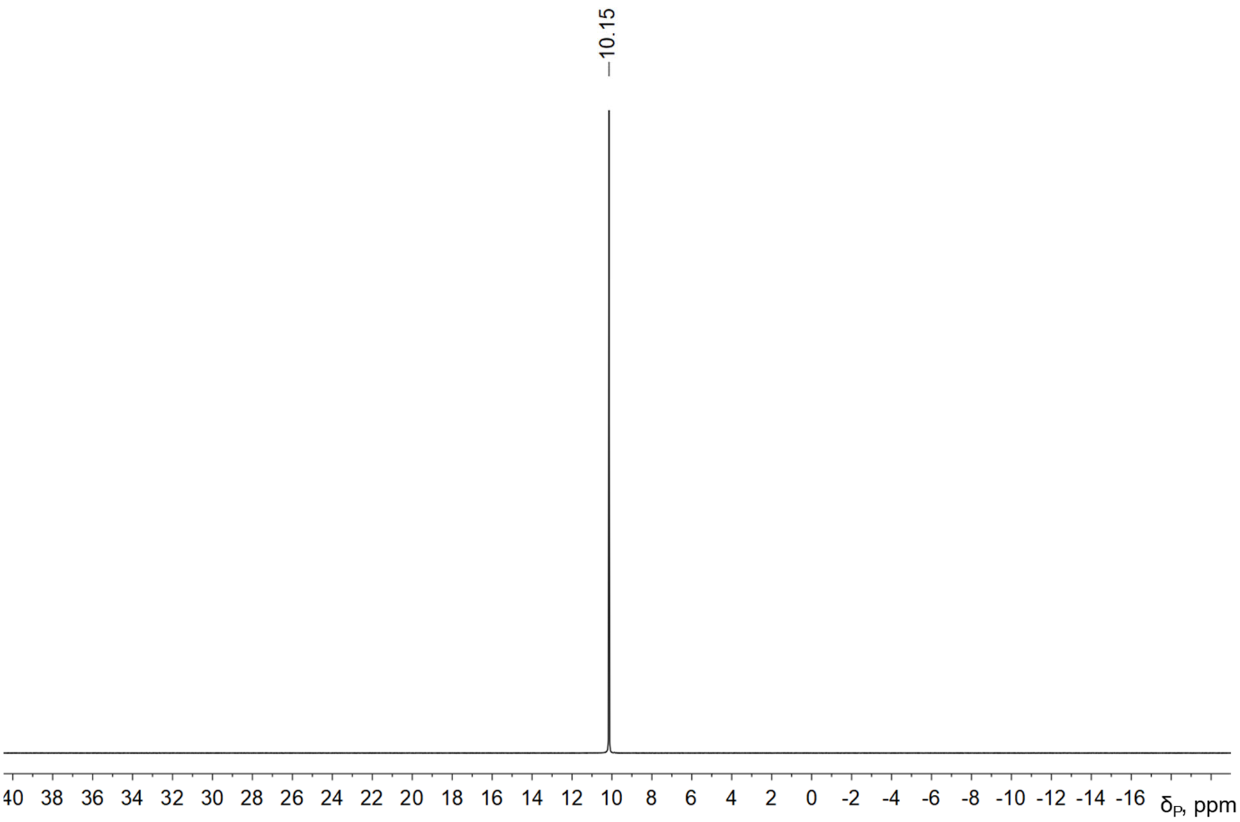

Figure S3. ^{31}P NMR spectra of hexa(p-methoxyphenoxy)cyclotriphosphazene, $CDCl_3$
